# Epigenetic origin of adaptive phenotypic variants in the human blood fluke *Schistosoma mansoni*

**DOI:** 10.1186/s13072-016-0076-2

**Published:** 2016-07-04

**Authors:** Sara Fneich, André Théron, Céline Cosseau, Anne Rognon, Benoit Aliaga, Jérôme Buard, David Duval, Nathalie Arancibia, Jérôme Boissier, David Roquis, Guillaume Mitta, Christoph Grunau

**Affiliations:** IHPE, Université de Perpignan Via Domitia (UPVD), 52 Avenue Paul Alduy, 66860 Perpignan Cedex, France; CNRS, UMR 5244, Interactions Hôtes-Pathogènes-Environnements (IHPE), 66860 Perpignan, France; CNRS, UPR1142, Institut de Génétique Humain (IGH), 34396 Montpellier, France; Technical University of Munich (TUM), Liesel-Beckmann-Str. 2, 85354 Freising, Germany; UMR BDR, INRA, ENVA, Université Paris Saclay, 78350 Jouy en Josas, France

**Keywords:** Epigenetics, Adaptive evolution, Compatibility polymorphism, *Schistosoma mansoni*

## Abstract

**Background:**

Adaptive evolution is not possible without the generation of phenotypic variants. The origin of these variations has been a central topic in evolutionary biology. Up to now, it was commonly accepted that standing genetic variation is the only cause of phenotypic variants. However, epigenetic information is emerging as a complementary source of heritable phenotypic variation that contributes to evolution. The relative importance of genetics and epigenetics in generating heritable phenotypic variation is nevertheless a matter of debate.

**Results:**

We used a host–parasite system to address this question. The human blood fluke *Schistosoma mansoni* can adapt rapidly to new intermediate snail hosts. The interaction between parasite and mollusk is characterized by a compatibility polymorphism illustrating the evolutionary dynamics in this system. The principal molecular marker for compatibility (infection success) is the expression pattern of a group of polymorphic mucins (*Sm*PoMuc) in the parasite. We show here that chromatin structure changes as the *Sm*PoMuc promoters are the cause for *Sm*PoMuc transcription polymorphism leading to phenotypic novelty and increase in infection success, i.e., fitness.

**Conclusion:**

We establish that epigenetic changes can be the major if not only cause of adaptive phenotypic variants in *Schistosoma mansoni*, suggesting that epimutations can provide material for adaptive evolution in the absence of genetic variation in other systems. In addition, our results indicate that epidrugs can be used to control parasite development but also parasite evolution.

**Electronic supplementary material:**

The online version of this article (doi:10.1186/s13072-016-0076-2) contains supplementary material, which is available to authorized users.

## Background

Adaptive evolution relies on the generation of heritable phenotypic variants on which selection can act. The origin of variation has puzzled researchers since Darwinian times [1]. There is now a relative broad consensus that genotype environment interactions represent the major, if not exclusive, source of phenotypic novelty. However, this view has been challenged by theoretical considerations [2–5], and it has been suggested that a substantial part of variability is the result of variations in the epigenetic component of the genome [6, 7]. A concept emerges in which upon environmental changes, the epigenetic (“low-fidelity”) system permits a population to generate new phenotypes while keeping genetic information invariant. This allows for exploring the fitness landscape after an environmental change and will “buy time” for this population. If the new environment persists, genetically encoded phenotypic variants could emerge and the new phenotype could be genetically assimilated. Heritability of epialleles has been clearly established for numerous examples (e.g., [8, 9]), and several epigenetically encoded phenotypic characters were described (e.g., [10, 11]). However, none of them is adaptive in the sense that the phenotype provides a fitness gain and is favored by selection under ecologically realistic conditions. It remains therefore an open question whether epigenetic inheritance contributes significantly to adaptive evolution. We addressed this question choosing a metazoan host/parasite system. Selective pressures are strong in these systems, evolution is fast and effective population sizes are small. We provide here, to the best of our knowledge, for the first time experimental evidence for an epigenetically encoded adaptive phenotype.

The parasite/host system we used is the interaction between *Schistosoma mansoni* and its intermediate host *Biomphalaria glabrata*. *S. mansoni* is a parasite of humans and causes intestinal schistosomiasis, the second most important human parasitic disease after malaria [12]. The life cycle requires passage through two obligatory hosts, the freshwater snail *Biomphalaria spp.* where the parasite multiplies asexually, and human or rodents as definitive hosts for sexual reproduction [13]. The snail/schistosome interaction is characterized by a phenomenon called compatibility polymorphism [14], i.e., specific strains of *S. mansoni* can only infect specific strains of *B. glabrata*, while others cannot be infected [15]. These incompatible snail strains are, however, not resistant to *S. mansoni* since they can be infected by other strains of the parasite. We had earlier identified *S. mansoni* polymorphic mucins (*Sm*PoMucs) as key molecular markers for compatibility [16, 17]. For several reasons, these proteins appear to be essential for the parasites to penetrate into the snail: (1) *Sm*PoMucs are expressed only in the snail infecting miracidia where they are excreted from the apical gland of the larvae. (2) *Sm*PoMucs present the strongest qualitative and quantitative differences between compatible and incompatible strains on the level of the proteome. Their exact function remains unknown but their amino acid sequence and glycosylation level [18] suggests that and (3) they form mucus that facilitates penetration through the snail epidermis. (4) They are recognized by the immune receptors of the invertebrate host [19] which triggers probably elimination of sporocysts of incompatible *S. mansoni* strains. *Sm*PoMucs are encoded by a multigene family that comprises at least 10 genes, organized in four clusters on the genome. These genes belong to a class of genes that are probably specific to Platyhelminthes and are called micro-exon genes (MEGs) [20]. The unusual structure of MEGs allows parasites to generate a “controlled chaos” of a polymorphic *Sm*PoMuc protein repertoire from a small number of genes using transcriptional, post-transcriptional and post-translational mechanisms [17]. We recently demonstrated that transcriptional control is based on epigenetic mechanisms [21]. We reasoned therefore that the compatibility phenotype would be a suitable model to disentangle the effects of genetically and epigenetically heritable components on adaptive traits. We applied a classical pedigree study and used two pure lines of *S. mansoni*: the Brazilian *Sm*BRE strain and the Guadeloupian *Sm*GH2 strain with their sympatric Brazilian snail strain *Bg*BRE and the Guadeloupian strain *Bg*GUA, respectively. Both strains show strong differences in compatibility (measured by prevalence and intensities) toward the same reference *B. glabrata* strain [15]. As mentioned above, *Sm*PoMucs are the key elements in the compatibility polymorphism [16], and expression of this gene family is regulated by epigenetic mechanisms [21, 22]. We therefore investigated expression of these genes and studied chromatin structure and DNA sequence in the promoter regions. We show that the compatibility phenotype and *Sm*PoMuc expression follow non-Mendelian segregation, that epialleles and alleles of *Sm*PoMuc do not cosegregate, and that treatment with an epimutagen modifies *Sm*PoMuc expression and increases fitness of the treated parasite larvae. Taken together our results indicate that not only *Sm*PoMuc expression but also infection success are at least partially under epigenetic control, thus providing an example for an epigenetically encoded adaptive trait.

## Results

### Inbred *S. mansoni* strains used in the study are pure lines

**Fig. 1:**
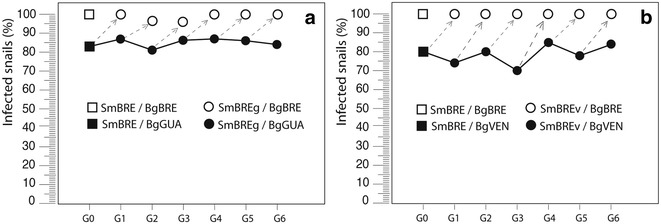
Compatibility of the *Sm*BRE schistosome strain with its homopatric snail host *Bg*BRE and two heteropatric snail strains, *Bg*GUA and *Bg*VEN: **a** *Sm*BREg corresponds to the *Sm*BRE strain passed for 6 generations (G1–G6) on *Bg*GUA snails; **b** *Sm*BREv corresponds to the *Sm*BRE strain passed for 6 generations (G1–G6) on the *Bg*VEN snails. At each generation, compatibility of the *Sm*BREg (**a**) and *Sm*BREv (**b**) was tested on the original *Bg*BRE snail strain

Our laboratory maintains the life cycle of currently five inbred strains of *Schistosoma mansoni* and their corresponding sympatric *Biomphalaria glabrata* host strains. These strains show a specific heritable reaction norm in their compatibility with *B. glabrata* strains [15]. Every strain possesses also a specific heritable pattern of *Sm*PoMuc so that we can “fingerprint” them by Western blots. To investigate whether the capacity to infect allopatric hosts can be selected for, we performed infections in allopatric combinations *Sm*BRE/*Bg*GUA and *Sm*BRE/*Bg*VEN and measured compatibility. The *Sm*BRE strain was originally sampled in Recife, Brazil, in the 1960s. It was provided to our laboratory in 1975 by Pr. Y. Golvan (Faculté de Médecine de Paris—Saint Antoine) and since then has been maintained in its sympatric intermediate host strain *Bg*BRE. *Bg*BRE is also from Recife and was also acquired in 1975. The allopatric mollusk strain *Bg*GUA originates from the town of Dans Fond and arrived at our laboratory in 2005. Snail strains *Bg*VEN were originally sampled in Venezuela. Prevalence (percentage of infected snails) is roughly 80 % in the allopatric combinations (*Sm*BRE on *Bg*GUA or *Bg*VEN) compared to the sympatric combination (*Sm*BRE on *Bg*BRE) where it is 100 % and this without significant variations for the six generations (Fig. 1a, b). Whatever the generation, offspring of both Schistosoma lines passed through allopatric snail hosts showed the same compatibility phenotype toward the original sympatric *Bg*BRE snail strain (100 %, Fig. 1a, b). Similar results were obtained for another *S. mansoni* strain, *Sm*GH2, hence fewer generations were followed. *Sm*GH2 was collected in 1983 from eggs shed by a patient in the hospital of Pointe-à-Pitre. Since then it was maintained in the laboratory in its sympatric snail host *Bg*GUA. About 60 % of snails are infected in the sympatric (*Sm*GH2 on *Bg*GUA) combination and less than 5 % in the allopatric combination (*Sm*GH2 on *Bg*BRE). Neither a gain of compatibility with the allopatric host nor a loss of compatibility with the sympatric host was detected. This means that the compatibility character cannot be selected for, i.e., offspring from allopatric combinations shows the same reaction norm as the original population. Therefore, even though the used strains are not clonal [23], they are pure lines in the sense of classical genetics.

### *Sm*PoMucs are involved in an early stage of host/parasite interaction

In order to infect its host, a parasite must find the host, adhere to the surface, penetrate and finally evade immune response inside the host. *Sm*PoMucs could be involved in any of these processes, but their constitutive expression in the apical glands of miracidia suggested involvement in the early steps, i.e., adherence and penetration. We reasoned that if this would be true, then bypassing penetration by transfer of sporocysts into snails would lead to no difference in prevalence between allopatric and sympatric combinations. Our results show that there is indeed no difference in the development of *S. mansoni* after transfer into the allopatric (*Sm*BRE in *Bg*GUA) *vs* the sympatric combination (*Sm*BRE in *Bg*BRE). In other words, if *Sm*BRE arrives inside the *Bg*GUA snail it behaves there as in a sympatric host (Additional file 1). This shows that when infecting allopatric *Bg*GUA snails, the limiting step is penetration and not sporocyst SpI development, and lends further support to the hypothesis that *Sm*PoMucs are involved in penetration or very early transformation steps.

### Strain hybrids express more *Sm*PoMuc variants than each parent

**Fig. 2:**
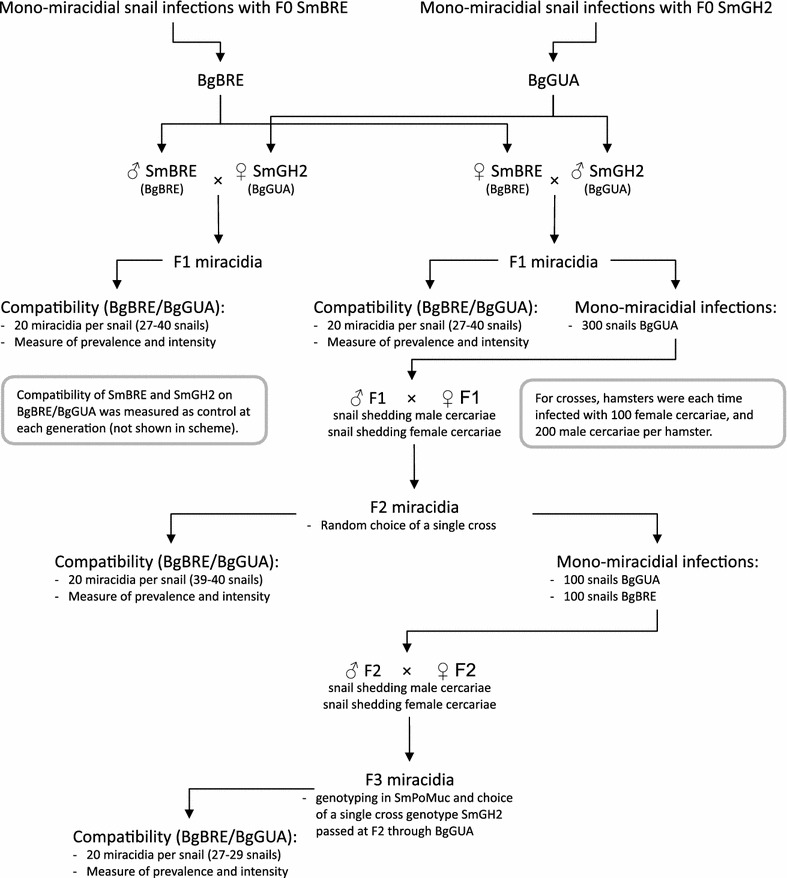
Crossing scheme for pedigree study. After monomiracidial infection of their sympatric hosts in F0, crosses within each generation were produced in each generation until F3

**Fig. 3:**
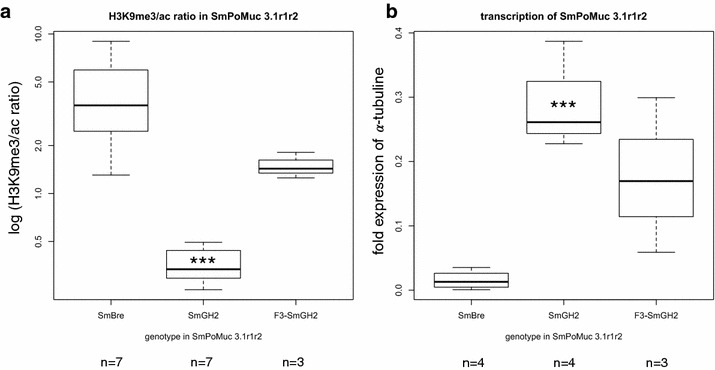
*Boxplot* of chromatin structure and transcription level in the promoter region of *Sm*PoMuc3.1 (r1r2). Two technical replicates for each biological replicate (*n*). **a** ChIP H3K9me3 to H3K9ac ratio at the promoter of *Sm*PoMuc 3.1(r1r2). **b** Relative transcription of *Sm*PoMuc 3.1(r1r2) compared to alpha-tubulin measured by RT-qPCR. ***indicate statistical significant differences according to ANOVA followed by Dunnett’s *post hoc* test to F0 *Sm*BRE that was defined as reference (ANOVA *F*_2,8_ = 13.128; *p* = 0.003 for transcription and *F*_2,14_=7.26; *p* = 0.007 for H3K9me3/ac ChIP ratio). Only *Sm*GH2 is significantly different in chromatin structure and transcription level from the reference. Genotype *Sm*GH2 of the F3 is similar (not different) to reference genotype F0 *Sm*Bre in terms of chromatin structure and transcription. For additional pairwise comparison we used Students T-Test. We found for H3K9me3/ac ratio (**a**) t = 10.045, 8 df, *p* < 0.0001, i.e. there is statistically significant difference between SmGH2 and F3-SmGH2. For transcription (**b**) the difference between SmGH2 and F3-SmGH2 is statistically not significant (t = 1.514; 5 df; *p* = 0.1904) since variance is strong. Nevertheless, there is a clear shift of transcription in F3-SmGH2 towards the lower level of transcription observed in SmBRE

**Fig. 4:**
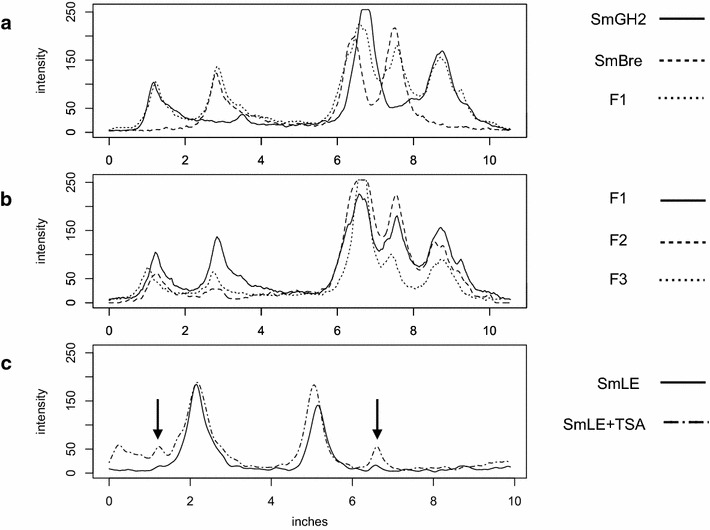
Expression profiles of *Sm*PoMucs. Western blot experiments were performed using a standardized method on proteins extracts from 1000 miracidia. Image was analyzed and grayscale (intensity on *y* axis) indicates protein abundance. X-axis migration distance in inches. **a** *Sm*PoMucs expression in F0 (*full and dashed lines*) and F1 (*dotted line*). F1 profile corresponds almost perfectly to the combination of both F0. **b** Comparison of *Sm*PoMucs expression in F1, F2 and F3. Global profile remains constant but 3 major bands decrease in intensity. **c** *Sm*PoMucs expression in *Sm*LE with (*dot-and-dash line*) and without TSA (*full line*) treatment. After TSA treatment, latent variants are now expressed (indicated by *arrowheads*)

We decided to introduce diversity by generating crosses between *Sm*BRE and *Sm*GH2. Thirty five to fifty snails from each strain (*Bg*BRE and *Bg*GUA) were exposed individually to 20 miracidia. Prevalence and intensity (number of mother sporocysts spI per snail) were measured. At each step, miracidia were set aside to perform molecular biology analyses. The experiments were carried out with miracidia obtained from *Sm*BRE × *Sm*BRE and *Sm*GH2 × *Sm*GH2 crosses, F1 miracidia obtained from reciprocal crosses of *Sm*BRE × *Sm*GH2, F2 miracidia were obtained from F1 × F1 and F3 miracidia obtained from crosses of two different clonal populations of F2 cercariae. The experimental scheme is summarized in Fig. 2. Quantitative RT-PCR shows that *Sm*PoMuc 3.1(r1r2) is on average 18.3-fold more transcribed in *Sm*GH2 F0 than in *Sm*BRE F0 strains (*n* = 4), and ChIP indicates a 12.05-fold higher acetylation of H3K9 in *Sm*GH2 F0 compared to *Sm*BRE F0 (*n* = 7) (Fig. 3). This confirms our earlier results that major control of expression of these genes occurs at the transcriptional level [21]. The hybrid lines F1 and F2 show higher transcription levels compared to *Sm*BRE F0 parent (data not shown). In the F3 generation, *Sm*PoMuc 3.1(r1r2) transcription is statistically not anymore different from *Sm*BRE F0 (Fig. 3). We wondered if such differences in transcription levels concerned also other genes and tested 14 arbitrarily chosen protein coding genes and transcribed repeats by qRT-PCR (genes and primers in Additional file 4). In none of the cases, we detected significant differences in transcription between the 4 generations (data not shown). We then used Western blots to test if *Sm*PoMuc transcripts are translated. *Sm*PoMucs proteins were detected using an antibody that recognizes the C-terminal conserved sequence by Western blot [19]. Proteins were extracted from 1000 miracidia for each condition. We knew already that although there is very little nucleotide differences in the promoter regions of *Sm*PoMucs family genes [21], the expression profiles between the parental strains *Sm*BRE F0 and *Sm*GH2 F0 were different [19], and we confirmed this here by Western blot (Fig. 4a). F1 hybrid miracidia express all bands of F0 parents, leading to a combined profile that corresponds almost perfectly to a superposition of *Sm*GH2 and *Sm*BRE parental profiles (Fig. 4a). In F2 and F3, we still find all bands of F0 parents even if their proportions change (Fig. 4b). Western blots were repeated with three F3 clones, and regardless of their genotype we always found the combined pattern of *Sm*PoMuc expression. Since *Sm*PoMucs are key markers for host–parasite compatibility, we expected the hybrids with a combined *Sm*PoMuc profile to show changes in compatibility. We therefore investigated their capacity to infest different snail strains.

### F3 Hybrids show higher fitness than the parents

**Fig. 5:**
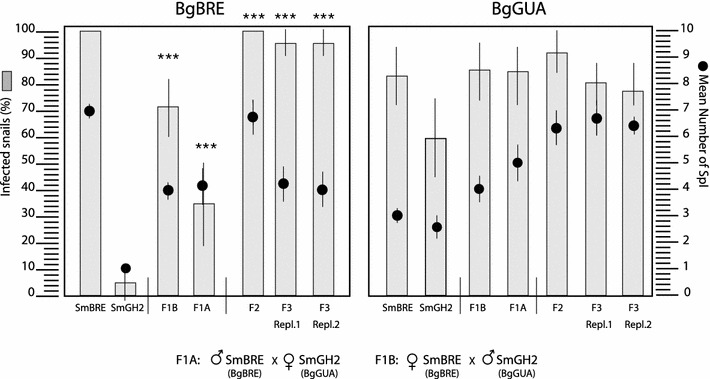
Prevalence and intensity of *S. mansoni* infection in *B. glabrata* snail strains. Prevalence is expressed as % of infected snails, intensity as mean number of SpI sporocysts per snail. ***indicate were prevalences and intensities increase significantly (Fisher’s exact test, *p* < 0.0001) compared to *Sm*GH2. They are not different from *Sm*BRE F0 (*p* < 0.05). Each individual snail was exposed to 20 miracidia

Prevalences in *Bg*BRE are 96–100 % for *Sm*BRE F0 and 4–6 % for *Sm*GH2 F0. Prevalences increase significantly (Fisher exact test, *p* < 0.0001) compared to *Sm*GH2 in F1, F2 and F3 to reach maximal values in F2 and F3 (Fig. 5***). Prevalences are statistically not different from *Sm*BRE F0 in all hybrid generations, thus we consider them to be similar. Intensity in *Bg*BRE is 7.1 ± 0.25 (mean ± SE) for *Sm*BRE (*n* = 34) and 1 ± 0 for *Sm*GH2 (*n* = 50). Similar to prevalence, intensity values increase significantly compared to *Sm*GH2 in F1, F2 and F3 (Fig. 5).

In *Bg*GUA snails, prevalences are 80–83 and 55–60 % for parental miracidia *Sm*BRE F0 and *Sm*GH2 F0, respectively. F1–F3 have infectivities that are statistically not different from *Sm*BRE F0 (*p* < 0.05, Fisher exact test) but higher than those of the *Sm*GH2 F0 parents (Fig. 5). For infection intensities of F0–F3 on *Bg*GUA, there is no significant difference between *Sm*BRE and *Sm*GH2 F0 parents, but we observe an increase in intensity values across generations F1–F3 (Fig. 5). The measure of the superior performance, i.e., heterosis on intensity parameter was calculated following the formula ((Crossbred average − Parental average)/Parental average) × 100. On *Bg*BRE, the crossbred F1 intensity is roughly 40 % greater than the average parental intensity. Consequently, all crosses of the two different strains of the parasite *S. mansoni* show increasing infection success of hybrids over three generations, i.e., an increase in global fitness. Interestingly, while the *Sm*PoMuc expression (molecular) phenotype is additive, compatibility phenotypes in F2 and F3 hybrids show uniparental phenotypic dominance of *Sm*BRE. Segregation of *Sm*PoMuc patterns and infection success is uncoupled, which is inconsistent with Mendelian genetic inheritance of a single locus or few loci. Consequently, we explored other mechanisms responsible for fitness increase. Since we had earlier shown that chromatin structure controls *Sm*PoMuc expression [21], we decided to study segregation of chromatin marks at the *Sm*PoMuc locus that shows the strongest chromatin structure differences and expression differences between *Sm*BRE and *Sm*GH2: *Sm*PoMuc 3.1 (r1r2).

### Chromatin structure in *Sm*PoMuc 3.1(r1r2) shows non-Mendelian segregation

*Sm*PoMuc promoter sequences do not have methylated cytosines [21]. Therefore, we focused our analysis on histone modifications in the promoter regions by native chromatin immunoprecipitation (N-ChIP) using two different antibodies that recognize histone H3 acetylated on lysine 9 (H3K9ac) and histone H3 tri-methylated on lysine 9 (H3K9me3). ChIP was followed by qPCR analysis to quantify the immunoprecipitated DNA. Since acetylation and methylation at H3K9 are mutually exclusive, we express chromatin status here as ratio of H3K9me3/H3K9ac. *Sm*PoMuc 3.1(r1r2) promoter shows a very different chromatin structure between the two F0 parents *Sm*BRE and *Sm*GH2 (Fig. 3). This is correlated with differential expression of this gene between the two parents. In F3, chromatin structure is closer to the *Sm*BRE F0 parent (Fig. 3). The promoter regions of *Sm*PoMuc 3.1 (r1r2) contain 3 diagnostic SNPs between *Sm*BRE F0 and *Sm*GH2 F0. To identify the genotypes at *Sm*PoMuc 3.1 (r1r2), we PCR-amplified and sequenced the *Sm*PoMuc 3.1 (r1r2) region using DNA of miracidia from all generations *Sm*BRE F0, *Sm*GH2 F0, F1, F2 and F3. As expected, F1 and F2 hybrid populations are heterozygous with two different parental alleles. We then studied in more detail F3 clones homozygous for the *Sm*GH2 genotype. We found that the *Sm*PoMuc 3.1 (r1r2) *Sm*GH2 genotype had aquired in F3 an epigenotype that is statistically not different from *Sm*BRE F0. One might argue that this is part of a genome-wide change in chromatin structure following the hybridization event. We therefore investigated another locus for which we had earlier identified differences in H3K9ac enrichment between *Sm*BRE and *Sm*GH2 [24]: Smp_171100.

**Fig. 6:**
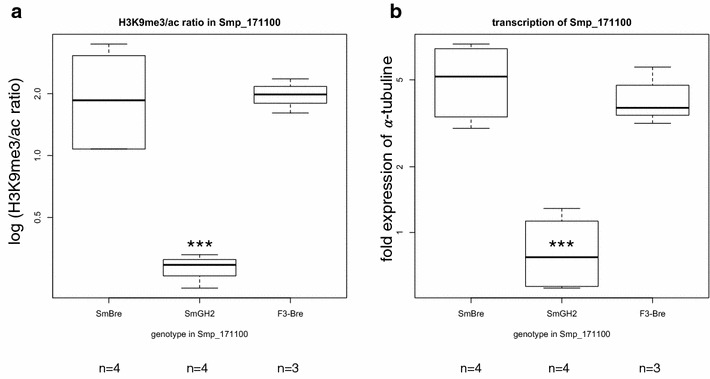
*Boxplot* of transcription level and chromatin structure in the gene body of Smp_171100. Two technical replicates for each biological replicate (*n*). **a** ChIP H3K9me3 to H3K9ac ratio. **b** Relative transcription compared to alpha-tubulin measured by RT-qPCR. ***indicate statistically significant differences as in Fig. 3 (ANOVA *F*_2,8_ = 7.56; *p* = 0.014 for transcription and *F*_2,8_ = 7.020; *p* = 0.017 for H3K9me3/ac ChIP ratio). Transcription level and chromatin structure co-segregate with the genotype

Smp_171100 codes for a putative M13 metallo-endopeptidases and is located on Schisto_mansoni.Chr_2 22,414,795–22,470,759. The function of the gene is not of particular importance here. The gene is not anymore listed in the latest version of the genome annotation, but we have confirmed the presence of a transcript by RT-PCR. We had shown earlier that chromatin differences exist in the gene body between *Sm*BRE and *Sm*GH2 [24], and we used them here as epigenetic markers for both strains. We confirmed our findings that *Sm*BRE F0 and *Sm*GH2 F0 show different H3K9me3/H3K9ac ratios. These differences in chromatin structure are correlated with transcription differences (Fig. 6). It should be noted that the difference is located in the body of the gene and not in the TSS, which explains the inverse relation of acetylation and expression. In the F3, the H3K9me3/H3K9ac ratio is not different from *Sm*BRE F0 (Fig. 6). We sequenced a region of the Smp_171100 gene sequence containing 6 SNPs between *Sm*BRE and *Sm*GH2. As expected and as in *Sm*PoMuc 3.1 (r1r2), F1 and F2 miracidial populations are heterozygous. The F3 clone that has the *Sm*GH2 alleles for *Sm*PoMuc 3.1 (r1r2)) is homozygous for *Sm*BRE in Smp_171100.

In conclusion, while in the *Sm*PoMuc 3.1(r1r2) locus, the *Sm*GH2 genotype had changed the epigenotype in the F3 (Fig. 3), in Smp_171100 we see a co-segregation of epigenotype and genotype (both are *Sm*BRE in F3). In other words, for the two loci in which to our knowledge there is a clear epigenetic difference between *Sm*BRE F0 and *Sm*GH2 F0, we distinguish two modes of chromatin heritability, one case of non-Mendelian inheritance (*Sm*PoMuc 3.1(r1r2)), and one case of Mendelian inheritance and cosegregation of genotype and epigenotype (Smp_171100).

### Pharmacological induction of chromatin structure changes leads to transcription of new *Sm*PoMuc variants and renders incompatible (avirulent) strain compatible (virulent)

All lines of evidence indicated that it was the H3K9me3/ac ratio that controlled specifically *Sm*PoMuc 3.1(r1r2) expression and that the change in chromatin structure resulted in new phenotypic variants based on new combinations of *Sm*PoMuc. This in turn led probably to the increase in infection success of the hybrids. If this was true, then perturbation of H3K9me3/ac ratio by other means should also lead to new *Sm*PoMuc variants and should have an impact on parasite–host compatibility. Locus-specific epigenetic engineering of *Sm*PoMuc is not yet feasible. We therefore opted for pharmacological treatment of *S. mansoni* eggs with the histone deacetylase inhibitor Trichostatin A (TSA). We had shown earlier that treatment with 20 µM TSA increased the number of alternative transcripts (number of polymorphic bands detected by RT-PCR) in *Sm*PoMuc but did not alter transcription of the abovementioned control genes [22]. Also in other systems, it was shown that TSA modulates transcription of around 5 % of genes and that genes can be up-regulated but also down-regulated by TSA [25–28]. In other words, TSA does not induce a global increase in transcription but will activate or inactivate certain loci only. We treated the *S. mansoni* eggs three times with 20 µM TSA before hatching. This dose have negligible cytotoxic effect [22]. For this part of the study, we used again two pure lines of *S. mansoni*: the Brazilian *Sm*LE strain (originally from Belo Horizonte) and *Sm*GH2 strain with the Brazilian snail *Bg*BAR2 (sympatric with *Sm*LE and also coming initially from Belo Horizonte). The *Sm*LE/*Sm*GH2—*Bg*BAR combinations were chosen because they have a very pronounced phenotype both in terms of compatibility (*Sm*GH2 has almost no capacity to infect of *Bg*BAR, but *Sm*LE is highly compatible) and in *Sm*PoMuc expression (only 2 strong bands in Western blots in *Sm*LE). Analysis of Western blots shows that TSA treatment leads to increased expression of *Sm*PoMuc protein variants in the miracidia that were latent in the untreated control (arrowheads in Fig. 4c), i.e., the already known transcription variation translates also into changes of the protein level. We subsequently investigated the compatibility phenotype using prevalences and intensities with mock treatment and with TSA treatment. *Bg*BAR snails were exposed individually to 20 miracidia. Using only the solvent ethanol as treatment, mean prevalences in *Bg*BAR are 95.65 % (number of exposed snails *n* = 23) for *Sm*LE, and 21.5 % for *Sm*GH2 (*n* = 97). Mean prevalences increased after treatment with TSA by 4–9 %: with TSA they reach 31.1 % (*n* = 103) for *Sm*GH2, and 100 % in *Sm*LE (*n* = 22). This is, however, not yet significant (Fisher exact test, *p* < 0.05). For intensity values, we detected an increase for *Sm*GH2 from 2.9 to 4.0 after TSA treatment, and for *Sm*LE from 2 to 9.55. This increase in individual infection success is significant (Fisher exact test, *p* < 0.05). Experiments were repeated four times by different experimenters (raw data provided as Additional file 2).

In conclusion, treatment with an epimutagenic agent does not only lead to changes in the *Sm*PoMuc expression patterns but also allows a parasite with low compatibility to increase its compatibility with a new snail host.

## Discussion

In parasite–host interactions, infection success can be used as an estimation for parasite fitness. It can be measured as number of infected host individuals (prevalence) and number of successful infection events per host individual (intensity). Using these parameters, we show here that after a hybridization event between two different *S. mansoni* strains, the offspring shows increased fitness. Increased fitness of hybrids is not surprising [29, 30], but in general such hybrid vigor or heterosis is attributed to increased genetic diversity in the offspring. This is probably also the case in *S. mansoni,* and host compatibility has necessarily a genetic basis. However, here we demonstrate that genetic diversity is not the only mechanism by which phenotypic diversity increases in hybrids. At least for the *Sm*PoMuc loci, epigenetic plays a more important role. This makes sense in light of the fact that nucleotide diversity between the two parental strains at these loci is extremely low [21]. One of the caveats of our study is that we did not address the question by which mechanism chromatin structure is modified. It could be that the *Sm*BRE locus is paramutagenic for *Sm*GH2. Paramutation is a heritable change in the expression of a paramutable allele, initiated by interaction in heterozygotes with a paramutagenic allele. Paramutations are meiotically stable and inherited in the absence of the inducing allele (e.g., [10]). Recent work places DNMT2, an enzyme that is able to methylate cytosine residues in tRNA, in the center of the paramutation pathway in animals [31] and a DNMT2 homologue (Smp_198180) exists in *S. mansoni* [32]. Another limitation in our approach comes from the non-specificity of the pharmacological induction of epimutations. We observed new *Sm*PoMuc combinations without changing the genetic background, and we tested a large number of reference genes that did not change transcription after TSA treatment, but we cannot exclude that transcription in other loci is modified. Nevertheless, we confirmed our earlier findings that epigenetic mechanisms control transcription of the *Sm*PoMuc genes and we show now that epigenetic information is indeed the origin of phenotypic novelty in these loci. The exact function of *Sm*PoMucs is still elusive; nevertheless, our earlier studies had firmly established *Sm*PoMuc variability as a key marker for compatibility between *S. mansoni* and its intermediate host [17, 19], and here, we show that *Sm*PoMucs are involved in the very early steps of infection (adherence, penetration and/or pre-Sp1 development). We conclude that strain hybridization and TSA treatment lead to changes in the epigenetic information that establish novel developmental trajectories leading to new and more phenotypic variants in the population. This allows the parasite population to explore the fitness landscape. If new matching phenotypes between parasites and mollusk host are produced, the latter can be infected, i.e., the phenotype is adaptive. This strategy might have evolved as a result of the combination of characteristics of the two hosts. Snail populations in endemic areas show very low prevalence (e.g., [33]), and it is often impossible to catch infected snail. This high selective pressure has led to local adaptation and compatibility with allopatric snail strains. Sexual reproduction of the parasite takes place within the vertebrate host, and when miraciadia are released they must immediately find a suitable snail host: at 25 °C, infection success declines rapidly 4 h after hatching and is zero after 12 h [34]. Non-migrating human hosts (e.g, school children) will shed eggs good for infection of sympatric snails. However, vertebrate hosts might also disperse (e.g., herdsmen) and parasite offspring might encounter allopatric snail hosts in this case. If the parasite cannot sense migration, a mixed stable/bet-hatching strategy would be best. However, if the parasite could sense migration of the host through dietary changes (e.g., different food, beverages, and starvation), modification of biorhythm (e.g., migration at night) or hormonal changes (e.g., stress), a switch from stable transmission to bet-hatching would be even more successful. The need of the parasite to receive triggers from the vertebrate host to complete its maturation is well documented and several hormones have been identified that alter parasite development (reviewed in [35]). In this sense, our TSA treatment might have mimicked events that trigger the switch between low and high phenotypic diversity. A schematic representation of this scenario is given in Additional file 3. Further work is needed to identify the biological trigger that leads to increased phenotypic variation in *S. mansoni* miracidia. Nevertheless, our findings fit already perfectly into theoretical models that predict such a function for the epigenetic information [2, 4] and are to the best of our knowledge the first experimental evidence for an epigenetic basis of adaptive evolution.

## Conclusion

We show here that histone modifications, i.e., changes in the epigenetic information can be a source of phenotypic variants in the parasite *S. mansoni*. These phenotypic variants are adaptive since they confer a higher fitness to the parasite by increasing its infection success in the intermediate mollusk host. It is conceivable that environmental clues trigger epigenetic variation and thus contribute to exploring the adaptive landscape. The genetic and molecular bases for the generation of epigenetic variants have not been investigated in this study.

## Methods

### Ethics statement

Our laboratory has permission A 66040 from both French Ministère de l’agriculture et de la pêche and French Ministère de l’Education Nationale de la Recherche et de la Technologie for experiments on animals and certificate for animal experimentation (authorization 007083, decree 87-848 and 2012201-0008) for the experimenters. Housing, breeding and animal care followed the national ethical requirements.

### Culture of *Schistosoma mansoni* parents and hybrids strains

We used in this part of the study different strains of *S. mansoni*: *Sm*BRE, *Sm*GH2 and *Sm*LE. Each strain was maintained in its sympatric mollusk *Biomphalaria glabrata* strain (*Bg*BRE, *Bg*GUA and *Bg*BAR2, respectively) as intermediate host and in hamsters (*Mesocricetus auratus*) as definitive host as described previously [36]. Three generations of hybrids of *Sm*BRE and *Sm*GH2 were produced as follows. Monomiracidial infections were performed within each strain of mollusk *Bg*BRE and *Bg*GUA. We obtained clonal populations of cercariae after 4 weeks. Sex was determined by PCR as described before [37, 38]. Strain hybrids were produced by infection of hamsters with 300 cercariae: 200 males from a clonal cercarial population and 100 females from another clonal population for each generation. Different combinations of parental cercariae were used for each generation in order to generate biological replicates. Three months later, eggs were collected from hamster livers and hatched in spring water. Miracidia were divided into three parts: (1) one part was used for life trait studies; (2) a second part was used for pedigree; and (3) a third part was concentrated by sedimentation on ice for 30 min and stored for molecular analysis at −20 °C.

### *Sm*BRE sporocysts transfer into *Bg*BRE and *Bg*GUA mollusks

*Sm*BRE miracida freshly hatched from a mouse liver were used to perform single miracidium infection of a dozen *Bg*BRE, as detailed in [39]. Thirty days later, mollusks were screened for presence of parasites. Two mollusks with a large number of sporocysts were selected as donors for sporocyst grafts.

Grafts were performed as described in [40, 41]. This technique allows to transplant secondary sporocysts from one mollusk to another. Grafted secondary sporocysts revert to primary sporocysts and then underdo normal development until cercariae emission. Large (10–12 mm) *Bg*BRE and *Bg*GUA mollusks were selected as receivers and anesthetized by incubating 4 h in spring water mixed with sodium pentobarbital at a final concentration of 1.2 mg/mL. Shells of mollusk donors were carefully removed with tweezers, and their digestive gland (where secondary sporocysts are located) was recovered. Explants of 1 mm^3^ containing 1–3 sporocysts were prepared and grafted (within an hour) in the cephalopedal sinus of receiver snails with a custom-made glass microneedle attached to a 1-mL syringe. A small incision of the tegument above the genital pore was made to reach the cephalopedal sinus. Grafted snails were maintained in normal growing conditions. Sporocysts from the first mollusk donor were grafted in 16 *Bg*GUA and 14 *Bg*BRE, and sporocysts from the second mollusk donor were transplanted in 17 *Bg*GUA and 15 *Bg*BRE.

Sixty days after transplantation, mollusks were screened for presence of parasites by searching for secondary sporocysts under a stereomicroscope and by looking for cercariae emission. Grafts were considered successful if both conditions were met. Fisher’s exact test for count data was used to compare between sympatric and allopatric sporocyst transfer success. The experiment was done in duplicates.

### Sex identification of cercariae

Four weeks after the monomiracidial infestation of mollusk for each generation *Sm*BRE F0, *Sm*GH2 F0, F1 and F2, four clonal cercariae were selected from each mollusk in order to identify the sex by PCR [37, 38]. After DNA extraction from single cercariae, PCR was performed using two pairs of primers, two control primers that amplify Rhodopsin [Smp_scaff001984 (49840–50016)] on male and female, and two female-specific primers that amplify SmWSPP2 [Smp_scaff002739 (2682–3046)] only on female (Additional file 4). If all four reactions delivered the same result, we considered sex as identified. We then chose male and female clones to infect hamsters and produce the next generation.

### Pharmacological induction by Trichostatin A treatment

After dissection of 4 infested hamsters (2 by strain), livers were collected and divided into two equal parts to compensate for a potential host bias. Two half livers for each strain *Sm*LE and *Sm*GH2 were incubated in 20 mL 150 mM NaCl and 0.1–0.3 % ethanol as control. For the treatment, two half livers for each strain *Sm*LE and *Sm*GH2 were incubated in 20 mL NaCl, and 20 µl of 20 mM Trichostatin A (TSA) (InvivoGen met-tsa-5) dissolved in ethanol was added two times at an interval of 12 h. Livers were incubated overnight at 4 °C. The next day, directly after grinding livers, another 20 µl of 20 mM TSA was added to the eggs from treated livers for *Sm*LE and *Sm*GH2. This strategy was chosen since TSA is known to be instable [42]. The final concentration of TSA was 20 µM in 150 mM NaCl and 0.1–0.3 % ethanol (solvent), and total treatment time was 16 h. Miracidia (non-treated *Sm*GH2, treated *Sm*GH2, non-treated *Sm*LE and treated *Sm*LE) were divided into two parts (1) for prevalence and intensity analysis and (2) for Western blot analysis.

### Compatibility of *S. mansoni* with mollusk hosts

Single mollusks (*n* = 30–50) were exposed to 20 miracidia each in 5 ml of spring water overnight at 24–25 °C. Prevalence was measured 15 days post-exposure, by determining the rate of infected mollusks over the entire mollusks that were exposed to parasites. The intensities were evaluated by counting the number of mother sporocysts that developed within infected mollusk as previously described [43]. For selection experiments, the *Sm*BRE strain was used for 6 generations on the *Bg*GUA and *Bg*VEN snail strains, respectively. Prevalences were determined at each passage. We named *Sm*BREg and *Sm*BREv these schistosome strains maintained on heteropatric snail hosts. At each generation, infectivity of *Sm*BREg and *Sm*BREv was tested *vis*-*à*-*vis* the original sympatric *Bg*BRE snail strain.

### Western blot

One thousand miracidia were individually collected, counted and incubated in 30 µl Lämmli buffer, 5 min at 99 °C. Fifteen µl were separated by electrophoresis through a 10 % SDS-PAGE gel and blotted on a nitrocellulose membrane (Trans-Blot turbo, Bio-Rad). The membrane was blocked with 5 % skimmed dry milk in TBST (TBS buffer containing 0.05 % Tween 20) for 1 h at room temperature and then incubated with the primary antibody “anti-*Sm*PoMuc” [19] diluted 1/500 in TBST for 90 min at room temperature. The membrane was then incubated with secondary antibody (peroxidase conjugated, purified anti-rabbit IgG) diluted 1/5000 in TBST for 1 h and washed three times with TBST. Finally, proteins were detected with a ChemiDoc MP Imaging system (Bio-Rad) using ECL reagents. Images were converted into 256 grayscales, and analysis was done with ImageJ [44].

### RNA extraction, reverse transcription and quantitative PCR

Messenger RNAs were isolated from 500 to 5000 miracidia from each strain using the Dynabeads^®^ mRNA DIRECT Micro Kit (Invitrogen). The samples were put directly into 100 µl lysis buffer at −80 °C and processed according to the manufacturer’s instructions. After washing, the samples were resuspended directly in 20 µl of DNase treatment mix (Ambion RNA by Life Technologies DNA-*free*). cDNA were synthesized from 13 µl of the total mRNA preparation, in a final volume of 20 µl using the RevertAid Premium First Strand cDNA Synthesis Kit by Thermo Scientific. Quantitative PCR analyses were carried out using 2.5 µl of cDNA diluted 1/10 in a final volume of 10 µl (1.5 µl H_2_O, 0.5 µM of each primer, 5 µl of master mix), using a LightCycler^®^ 480 Real-Time Instrument, and 2.5 µl of mRNA diluted 1/20 as negative control for the specific exon junctions amplifications. Alpha-tubulin (α-tub) was used as reference. Primer sequences are listed in Additional file 4. The following protocol was used: denaturation, 95 °C 10 min, amplification and quantification (45 cycles), 95 °C for 10 s, 60 °C for 5 s, 72 °C for 20 s; melting curve, 65–97 °C with a heating rate of 0.11 C/s and continuous fluorescence measurement, and cooling step to 40 °C. For each reaction, the crossing point cycle threshold (Ct) was determined using the “second derivate” method of the LightCycler^®^ 480 Software release 1.5.0. Reactions were carried out in duplicate then the mean Ct was calculated. The amplification of a unique band of each locus was verified by size separation on a LabChip GX capillary electrophoretic system.

### Chromatin immunoprecipitation (ChIP) followed by qPCR

Native chromatin immunoprecipitation was performed as described before [45]. The following antibodies against histone isoforms were used to precipitate chromatin in miracidia: Abcam anti-H3K9me3 (ab8898, Lot 733951) and Millipore anti-H3K9ac (07-352, Lot DAM1576933). Immunoprecipitated DNA was extracted by phenol/chloroform protocol and analyzed by quantitative PCR using specific primers for *Sm*PoMuc 3.1(r1–r2) promoter (primer sequences in Additional file 4) and for the Smp_171100. The amount of target DNA recovered in the immunoprecipitated fraction was quantified by calculating the percent of input recovery (% IR) normalized with the percent input recovery obtained with α-tubulin gene. The percent input recovery of the bound immunoprecipitated fraction for each amplicon was calculated by the following formula: % input recovery = 100 × *E*^(Ct (input) − Ct (IPBound))^. The percent background was calculated by the following formula: % background = 100 × *E*^(Ct (input) − Ct (*C*-Bound))^, where *E* is the primer efficiency designed to amplify the amplicon, Ct (IP_Bound_) is the Ct of the bound fraction obtained in the immunoprecipitated sample, Ct (*C*-_Bound_) is the Ct of the bound fraction obtained in the negative control (fraction without antibody) and Ct (input) is the Ct of the unbound fraction obtained in the negative control. It represents the quantity of chromatin that was used for the study minus the fraction that bound non-specifically to the protein A Sepharose beads. Finally, the ratios of the H3K9me3 to H3K3Ac for each gene was calculated using formulas (2^ct (α-tub)^/2^ct (*Sm*PoMuc 3.1(r1r2))^ and 2^ct (α-tub)^/2^ct (Smp_171100))^.

### DNA extraction, PCR amplification and Sanger sequencing

Genomic DNA for *Sm*BRE F0, *Sm*GH2 F0, F1, F2 and F3 miracidial population, was prepared by the incubation during 3 h at 55 °C with 500 µl of lysis buffer (20 mM Tris/Cl Ph 8; 1 Mm EDTA; 100 mM NaCl; 0.5 % SDS) and 0.15 mg of proteinase K. The samples were extracted twice with equal volumes of phenol/chloroform, followed by two extractions of equal volumes of chloroform. DNA was precipitated with equal volumes of isopropoanol/NaOAc (3 M, pH 5.2) at room temperature. After centrifugation and washing with 1 ml of 70 % ethanol, the pellet was dissolved in 50 µl of 1 mM Tris/Cl pH 8.

In order to determine whether the chromatin structure follows the Mendelian transmission or not (co-segregate with the allele or not), we focus specifically on sequences where we determined the chromatin structure by ChIP-qPCR for *Sm*BRE F0, GH2 F0, F1, F2 and F3 miracidial population. We identified 3 SNPs between *Sm*BRE F0 and GH2 F0 for *Sm*PoMuc 3.1(r1r2) promoter sequence and 6 SNPs for Smp_171100 sequence using Sequencer software. Primers including SNPs for the two loci were designed, and the regions were amplified by qPCR. For the *Sm*PoMuc 3.1(r1r2) promoter, qPCR was applied on a 9 kb PCR product containing the 2-kb region upstream of the transcription start site (TSS) [21], and for Smp_171100 sequence, qPCR was applied directly on genomic DNA for *Sm*BRE F0, *Sm*GH2 F0, F1, F2 and F3 miracidial population. qPCR products were sequenced using Eurogentec facilities (http://www.eurogentec.com/life-science.html). We checked and aligned nucleotide sequences manually using Sequencer and BioEdit softwares.

### Statistical analysis

Fisher’s exact test was used to compare prevalences and T test to compare intensities. For the statistical differences in the amount of transcripts, we used ANOVA with Dunnett’s *post hoc* test using the parental strain *Sm*BRE as control group.

## Additional files

10.1186/s13072-016-0076-2 Infection success after sporocyst transfer. *Sm*BRE sporocysts succeed to infect the two strains of mollusks *Bg*BRE and *Bg*GUA and the vertebrate host.
10.1186/s13072-016-0076-2 TSA treatment results. This table contains results of all TSA experiments.
10.1186/s13072-016-0076-2 Schematic representation of compatibility polymorphism and influence of changes in the epigenotype. On the left a high compatibility situation: the hypothetical inbred *S. mansoni* strain “white” can infect successfully the *B. glabrata* strain “MOSTLY WHITE” because most snails are compatible “white.” However, *Sm*WHITE cannot infect *B. glabrata* “dark” snails. On the right, the incompatible situation in which the same *Sm*WHITE strain is exposed to a *Bg*DARK strain. None of the *Bg*DARK can be infected by *Sm*WHITE. If *Sm*WHITE is treated with TSA, new phenotypic variants develop. Some of them (e.g., *Sm*BLACK) are also incompatible with the snail hosts; others can infect (e.g., *Sm*GRAY). By increasing the phenotypic variants in the inbred strain through epimutations, the reaction norm of the strain becomes larger and previously incompatible hosts can be infected.
10.1186/s13072-016-0076-2 Primers used in this study.

## Abbreviations

*Sm*PoMuc: *Schistosoma mansoni* polymorphic mucins
*Sm*BRE: *Schistosoma mansoni* strain Brazil BRE
*Sm*LE: *Schistosoma mansoni* strain Brazil LE
*Sm*GH2: *Schistosoma mansoni* strain Guadeloupe GH2
*Bg*BRE: *Biomphalaria glabrata* strain Brazil BRE
*Bg*GUA: *Biomphalaria glabrata* strain Guadeloupe GUA
*Bg*BAR: *Biomphalaria glabrata* strain Brazil BAR
ChIP: chromatin Immunoprecipitation
H3K9ac: histone H3 acetylated on lysine 9
H3K9me3: histone H3 tri-methylated on lysine 9
TSA: trichostatin A
