# Supplementary material for: Epigenetic origin of adaptive phenotypic variants in the human blood fluke Schistosoma mansoni
Source: Epigenetics Chromatin. 2016 Jul 4;9:27. doi: 10.1186/s13072-016-0076-2 (PMC4931705; doi:10.1186/s13072-016-0076-2)
Supplement: Supplementary file 1 — 10.1186/s13072-016-0076-2 Infection success after sporocyst transfer. SmBRE sporocysts succeed to infect the two strains of mollusks BgBRE and BgGUA and the vertebrate host. [file 13072_2016_76_MOESM1_ESM.docx]

Additional file 1 :

| *S. mansoni* BRE | *B. glabrata* BRE | | *B. glabrata* GUA | | Adult worms in vertebrate host | |
| --- | --- | --- | --- | --- | --- | --- |
|  | Infected | Not infected | Infected | Not infected | From *Bg*BRE | From *Bg*GUA |
| Replicate 1 | 1 | 13 | 6 | 10 | 17 | 30 |
| Replicate 2 | 5 | 10 | 9 | 8 | 171 | 405 |
| Total | 6 | 23 | 15 | 18 | 188 | 435 |
