# Supplementary material for: Epigenetic origin of adaptive phenotypic variants in the human blood fluke Schistosoma mansoni
Source: Epigenetics Chromatin. 2016 Jul 4;9:27. doi: 10.1186/s13072-016-0076-2 (PMC4931705; doi:10.1186/s13072-016-0076-2)
Supplement: Supplementary file 3 — 10.1186/s13072-016-0076-2 Schematic representation of compatibility polymorphism and influence of changes in the epigenotype. On the left a high compatibility situation: the hypothetical inbred S. mansoni strain “white” can infect successfully the B. glabrata strain “MOSTLY WHITE” because most snails are compatible “white.” However, SmWHITE cannot infect B. glabrata “dark” snails. On the right, the incompatible situation in which the same SmWHITE strain is exposed to a BgDARK strain. None of the BgDARK can be infected by SmWHITE. If SmWHITE is treated with TSA, new phenotypic variants develop. Some of them (e.g., SmBLACK) are also incompatible with the snail hosts; others can infect (e.g., SmGRAY). By increasing the phenotypic variants in the inbred strain through epimutations, the reaction norm of the strain becomes larger and previously incompatible hosts can be infected. [file 13072_2016_76_MOESM3_ESM.pdf]

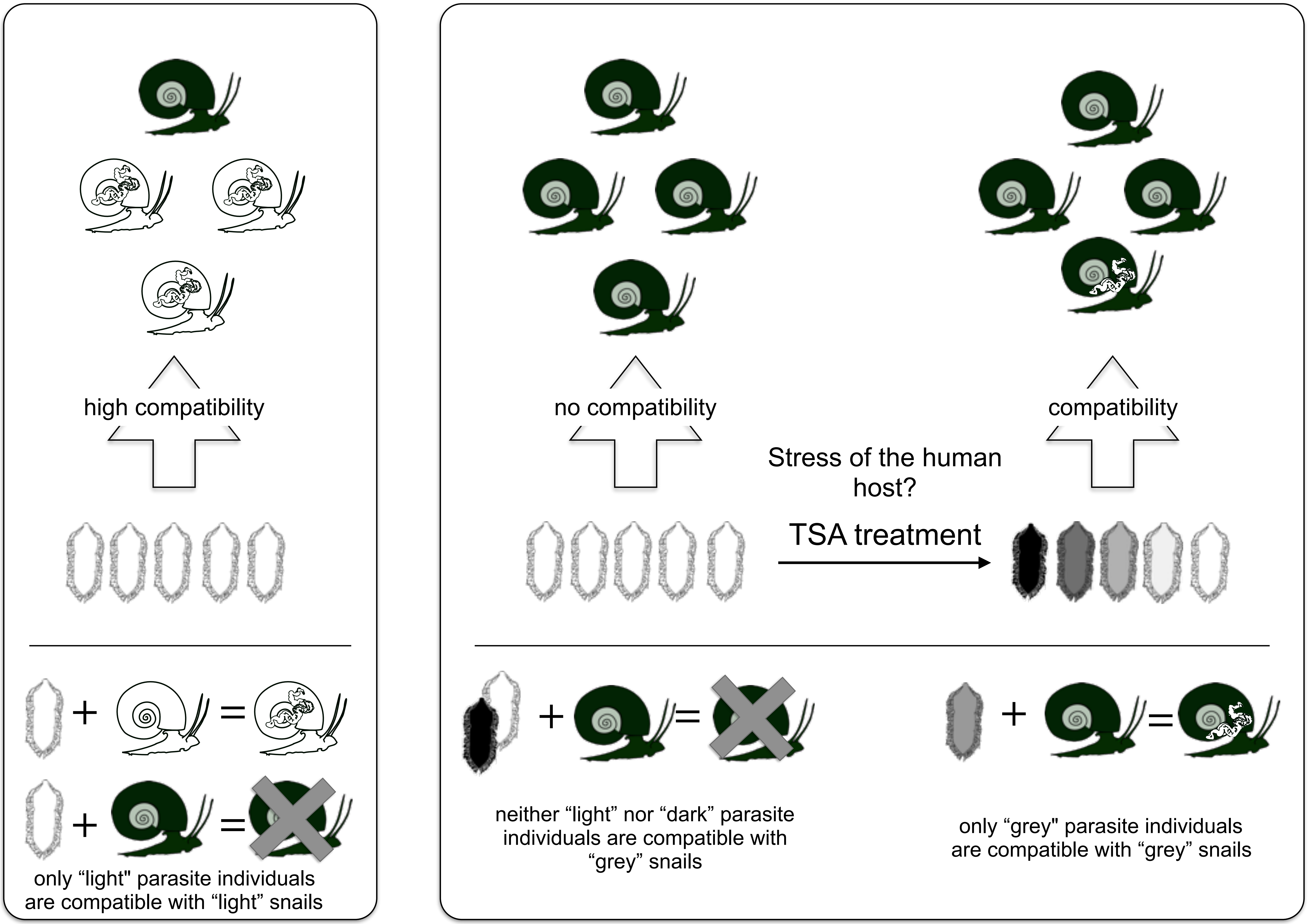
high compatibility
+
=
+
=
only “light" parasite individuals are compatible with “light” snails
no compatibility
compatibility
Stress of the human host?
TSA treatment
+
=
neither “light” nor “dark” parasite individuals are compatible with “grey” snails
+
=
only “grey" parasite individuals are compatible with “grey” snails
