# Supplementary material for: Epigenetic origin of adaptive phenotypic variants in the human blood fluke Schistosoma mansoni
Source: Epigenetics Chromatin. 2016 Jul 4;9:27. doi: 10.1186/s13072-016-0076-2 (PMC4931705; doi:10.1186/s13072-016-0076-2)
Supplement: Supplementary file 4 — 10.1186/s13072-016-0076-2 Primers used in this study. [file 13072_2016_76_MOESM4_ESM.docx]

# Additional file 4: Primers used in this study

**Primers used for sex identification of cercariae**

| **Target** | **Name** | **Forward (5’-3’)** | **Reverse (5’-3’)** | **Amplicon**  **length (bp)** |
| --- | --- | --- | --- | --- |
| **Rhodopsin** | Smp_scaff001984.F  Smp_scaff001984.R | GACGGCCACACTAAAG | AGTAAAATGGTCACTGCTAT | 191 |
| **SmWSPP2** | Smp_scaff002739.F  Smp_scaff002739.R | TGTTTCGAATTTCACACTT | CATTCACAGTTTGGCGAACA | 391 |

**Primers used for expression analysis by qPCR**

| **Target** | **Name** | **Forward (5’-3’)** | **Reverse (5’-3’)** | **Amplicon**  **length (bp)** |
| --- | --- | --- | --- | --- |
| **-tubuline** | aTub2.F  aTub2.R | TAGCGCACCATCGAAGCGCA | GCTTTCATGGTTGACAACGAGGCCAT | 65 |
| ***Sm*PoMuc 3.1(r1r2)** | URG/B.F  *Sm*PoMuc3 E2/E3.R | CGAACCAACAGGTGACCTCGC | CTGGAACTGTTGGTTCGCTC | 113 |
| **MSPP tot** | MSPPtot.F  MSPPtot.R | GAATTGGAATCGAAGGCG | TGTTCATCTTTGGGTAGTGT | 182 |
| ***Sm*PoMuc1** | smpomuc1.E8/9.F  smpomuc1.E12.R | CGAAAGTGCTTACATCGCTG | CGAATAGGCTTCTTTATGTTG | 190 |
| ***Sm*PoMuc2** | smpomuc2.E4.F  smpomuc2.E8.R | CCAGATCCACAACAGCACC | ACTTTCGACGTCATCAAGAAG | 240 |
| **GST** | GST.B.F  GST.B.R | CAAAGGCCTTGTACAACCAACTCGT | CGTTTCGCCACACATCGCCG | 97 |
| **GAPDH** | GAPDH.F  GAPDH.R | TGGCCGTGGAGCGATGCAAA | CGTCTGGTGTTGGGACGCGG | 122 |
| **R564 W5** | R=564.F  R=564.R | ACCGCGTCTCCCCTCTTCAGT | AGGGAGAGCGCACACCATGA | 137 |
| **R879 W4** | R=879.F  R=879.R | CGGTCATGGAGGCGTGGTCA | CTCGTCGTACCATGACAATCAGCA | 120 |
| **smPDI** | SmPDI.F  SmPDI.R | GGGATTTATCAAGGATACGGACTC | CACCAAGGAGCATACAGTTTGAC |  |
| **smProt1** | smProt1.F  smProt1.R | TGGCTATCGTAAATAAAGAAGGAA | TCAGCAATAGACCAATCACATTT | 72 |
| **cox 2** | Cox2.F  Cox2.R | AGATGGATGCGGTTCCAGGTCG | AGCATGACCCACACCGCATAAC | 104 |
| **Smp 152710.1** | Smp_152710.1A.F  Smp_152710.1A.R | acagctctagttgtcgaccaaacatc | acgcggtcaacgtaagggca | 132 |
| **Smp 054160** | Smp_054160A.F  Smp_054160A.R | cggacgcggacgtgctgaat | caggcaatcgtccgcctgga | 114 |
| **Smp 158110.1** | Smp_158110.1A.F  Smp_158110.1A.R | GCAAAGCTGGTGGTTTGGGGC | TGCATGACCTTCCTGTTCGTCAAG | 90 |
| **Spo/AF109180** | Spo4.F  Spo4.R | TGTGTCGTAGGTGCTATG | TGCCTAAATCTTCCTTCCG | 204 |
| **Ef1a** | Ef1a2.F  Ef1a2.R | AAACTGAAGGCTGAACGCGAACG | AGCCGCAACAATCAGCATTGCAC | 158 |

**Primers used for ChIP analysis by qPCR**

| **Target** | **Name** | **Forward (5’-3’)** | **Reverse (5’-3’)** | **Amplicon**  **length (bp)** |
| --- | --- | --- | --- | --- |
| **-tubuline** | ATub.F  ATub.R | AGCAGTTAAGCGTTGCAGAAATCA | TGACGAGGGTCACATTTCACCAT | 65 |
| ***Sm*PoMuc 3.1(r1r2)** | *Sm*PoMuc 3.1(r1r2) | ATGATTTTACGAGAGGTTCAGT | ggtgcactattcagcctagtca | 101 |
| **Metallopeptidase** | Smp171100.F  Smp171100.R | CCTGGAATGGAGAGCTCGTATGACA | GGAGTGTGGATTGAACGCGTCG | 115 |

**Primers used for PCR amplification and specific sequencing around SNPs**

| **Target** | **Name** | **Forward (5’-3’)** | **Reverse (5’-3’)** | **Amplicon**  **length (bp)** |
| --- | --- | --- | --- | --- |
| ***Sm*PoMuc 3.1(r1r2)** | exon2.F  exon1.R | TTCTTAGCACTACCCAAAGATGAAC | AGAGAATAATTTTCTTGTTCATTCTTC | 9000 |
|  | R1R2-SNP1.F  R1R2-SNP1.R | CACGTGTTGCAAAATAACCG | GAAATCCCGGAAGCACTGTA | 204 |
|  | R1R2-SNP2.F  R1R2-SNP2.R | CCGCAGACGAGTCCAATAGT | AGCAACCGATGATTTGCATT | 115 |
|  | R1R2-SNP3.F  R1R2-SNP3.R | TTTCCAAAACGTTGGACCAT | CGATTGTAAATTTGGAAACGAT | 170 |
|  | R1R2-SNP4-5.F  R1R2-SNP4-5.R | CATGAACAGGGAAATCACGA | AAAACCATGTTGTTCCGAGG | 127 |
| **Metallopeptidase Smp_171100** | Smp171100.F  Smp171100.R | CCTGGAATGGAGAGCTCGTATGACA | GGAGTGTGGATTGAACGCGTCG | 115 |
